# Impacts of microhabitat changes on wintering waterbird populations

**DOI:** 10.1038/s41598-017-14317-9

**Published:** 2017-10-24

**Authors:** Ye-Ai Zou, Bai-Han Pan, Hong Zhang, Ping-Yang Zhang, Yi Yao, Xiang-Kui Liu, Da-Li Gao, Yong-Hong Xie

**Affiliations:** 1 Key Laboratory of Agro-ecological Processes in Subtropical Regions, Chinese Academy of Sciences, Hunan, 410125 PR China; 2 Dongting Lake Station for Wetland Ecosystem Research, Institute of Subtropical Agriculture, Chinese Academy of Sciences, Changsha, 410125 PR China; 3 School of Life Science, Shanghai Key Lab for Urban Ecological Process and Eco–Restoration, East China Normal University, Shanghai, 200062 PR China; 4 University of Chinese Academy of Sciences, Beijing, 100049 China; 5 Administrative Bureau of Hunan East Dongting Lake National Nature Reserve, Yueyang, 414000 Hunan, PR China

## Abstract

Caisang Lake, a human-modified wetland, experienced dramatic habitat alterations from the planting of lotus and culturing of crab. Whether the Caisang Lake still maintains populations of wintering waterbirds is of great concern. Here, we compare the changes in waterbird populations before and after habitat alterations in Caisang Lake and assess the driving factors leading to the dramatic changes in waterbird populations. Results indicate that wintering waterbird populations were significantly impacted by altered forage availability, with species- and guild-specific responses. Dramatic habitat alterations from planting lotus caused significant declines in areas of native vegetation, mudflats, and water that may have caused associated declines in herbivores, insectivores, and fish-eating waterbirds, respectively. In contrast, the increased size of the lotus area appears to have led to an increase in omnivorous waterbirds. A food shortage, potentially caused by a large area of Caisang Lake being used for culturing crab, might be another cause of the observed decline in fish-eating waterbirds. This study demonstrates a powerful approach to systematically evaluate waterbird responses to wetland management policies. These findings are important as efforts are made to protect the wintering waterbirds from the effects of human intervention, particularly at other Ramsar wetlands.

## Introduction

Globally, natural wetlands are under heavy pressure and many have been transformed, degraded, or lost with the intensification of human activities and environmental changes^1,2^. Waterbird populations are being adversely threatened worldwide and have exhibited declining trends that are correlated with wetland habitat degradation and loss^3^. Dongting Lake, the second largest freshwater lake in China, is a Ramsar site and one of the eco-regions that is listed in the Global 200 as an important priority for the conservation of global biodiversity^4^; it has recently been recognized as the crucial wintering region in Yangtze River floodplain for hundreds of thousands of migratory waterbirds of the East Asian-Australasian Flyway, particularly for the Anatidae in Eastern China^5^. During the last several decades, climate change and the operation of dams have changed the hydrological regimes and thus the vegetation structure and succession^6^. Such dramatic changes may result in a lack of suitable wintering sites for wintering waterbird communities, which would affect their population and distributions in the natural wetlands of East Dongting Lake^7–10^.

How to provide high quality habitats for waterbirds and maintain their populations through effective wetland management is the crucial issue, especially when both the quality and quantity of natural wetlands have decreased^11,12^. Thus, artificial wetlands are performing a crucial role in biodiversity conservation worldwide^13–15^; however, it is still debated whether artificial wetlands are suitable alternatives to natural wetlands for waterbirds. Many studies recognize that artificial wetlands could potentially provide suitable habitats and serve as good alternative to natural wetlands for waterbirds during any stage of their life history, i.e. during breeding, migrating, stopover, and wintering stages^16,17^. However, debate surrounds whether artificial wetlands may only perform some but not all the functions of natural wetlands, so the question remains if artificial wetlands can replace the conservation value of natural wetlands in supporting greater numbers of waterbirds (species and abundance)^15,18–20^. This debate continues.

One example of human-modified artificial wetlands (Caisang Lake) lies to the north of the natural wetlands of East Dongting Lake. Previous studies indicate that Caisang Lake could be used as foraging habitat for wintering waterbirds, particularly for the lesser white-fronted goose (a herbivorous species), especially when food shortages occurred in the neighbouring natural East Dongting Lake wetlands^7,21,22^. Unfortunately, Caisang Lake suffered dramatic habitat alterations in 2013/2014 with the planting of lotus (*Nelumbo nucifera*) and culturing of crab (*Eriocheir sinensis*); this resulted in a sharp decline in the areas of vegetation, mudflats, and water habitats but a significant increase in the areas of lotus (details are discussed further in the Results section). However, it remains largely unknown whether, after the habitat alterations, the wetlands of Caisang Lake might still be used as foraging or roosting habitats to maintain wintering waterbird populations; uncertainty also remains about the crucial driving factors in the habitat that led to dramatic changes in waterbird populations in Caisang Lake.

Although both climate and food availability are crucial for waterbird populations and distributions^6,23,24^, finding sufficient food resources during non-breeding periods is, in particular, a major challenge for migratory birds^25^. Decline in food availability during non-breeding periods could lead to a sharp decline in waterbird populations^26,27^. Studies on avian community dynamics in response to habitat changes have shown that responses are mixed and affected by avian guild composition and structure^8,28^. We hypothesize that drastic habitat alterations, leading to a sharp decline of areas of vegetation, mudflats, and water habitats but an increase in area covered by lotus, might have negatively changed food availability for wintering waterbirds. In this way, drastic habitat alterations at Caisang Lake may have affected significant changes in the local waterbird populations. Moreover, the driving factors that can lead to dramatic changes in waterbird populations may differ among foraging guilds and individual species, due to their distinct feeding requirements.

We aim to test this hypothesis by (1) comparing the changes in waterbird populations at community, foraging feeding guild, and species levels before and after habitat alterations in Caisang Lake, and (2) analysing the relationships between changes in waterbird communities and the environmental variables during the 2003/2004–2016/2017 winters.

## Results

### Changes in Waterbird Habitats

According to the accuracy assessment of individual classifications (vegetation, water, mudflat, and lotus habitats) with a standard error matrix (confusion matrix)^29^, the overall accuracy of classifications (2004–2017, using reference data from GoogleEarth images) was greater than 88%. In contrast, the kappa statistic values for the same classifications were greater than 0.9.

**Figure 1:**
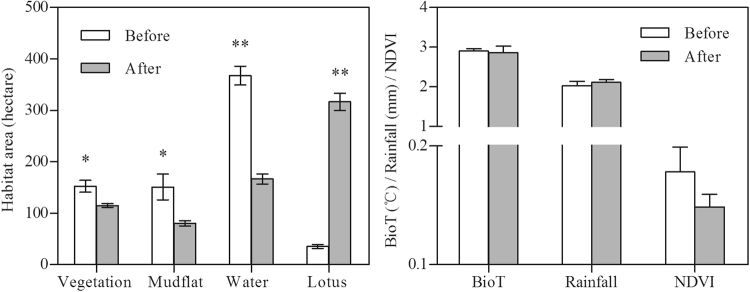
Changes in the environmental variables before and after habitat alterations. Error bars represent standard error (SE). Statistically significant values are represented by asterisks: *p < 0.05, **p < 0.01.

The waterbird habitats at Caisang Lake suffered dramatic alterations in 2013. Specifically, after 2013, the areas of vegetation, mudflats, and water habitats exhibited significant declines (vegetation area, t = 3.01, df = 8, p = 0.024 < 0.05; mudflat area, t = 2.78, df = 8, p = 0.036 < 0.05; water area, t = 7.35, df = 8, p < 0.001; Fig. 1), but the area of lotus habitat significantly increased (t = −51.01, df = 8, p < 0.001; Fig. 1). However, no significant differences were observed in the growth status of the vegetation (NDVI), BioT, or rainfall after the habitat alterations in 2013 (t-test, all p > 0.05; Fig. 1).

### Waterbird Population Dynamics

A total of 55,478 individuals, which comprise 7 orders, 11 families, and 52 species, were recorded in Caisang Lake in the wintering seasons between 2003/2004 and 2016/2017. Twelve rare species^30^ were observed (Table S1); of these, one species (oriental white stork *Ciconia boyciana*) is listed as endangered, five species are vulnerable (lesser white-fronted goose *Anser erythropus*, swan goose *A. cygnoides*, hooded crane *Grus monacha*, common pochard *Aythya ferina*, dalmatian pelican *Pelecanus crispus*), and six species are near-threatened (falcated duck *A. falcata*, ferruginous duck *A. nyroca*, northern lapwing *Vanellus vanellus*, black-tailed godwit *Limosa limosa*, bar-tailed godwit *L. Lapponica*, Eurasian curlew *Numenius arquata*).

**Figure 2:**
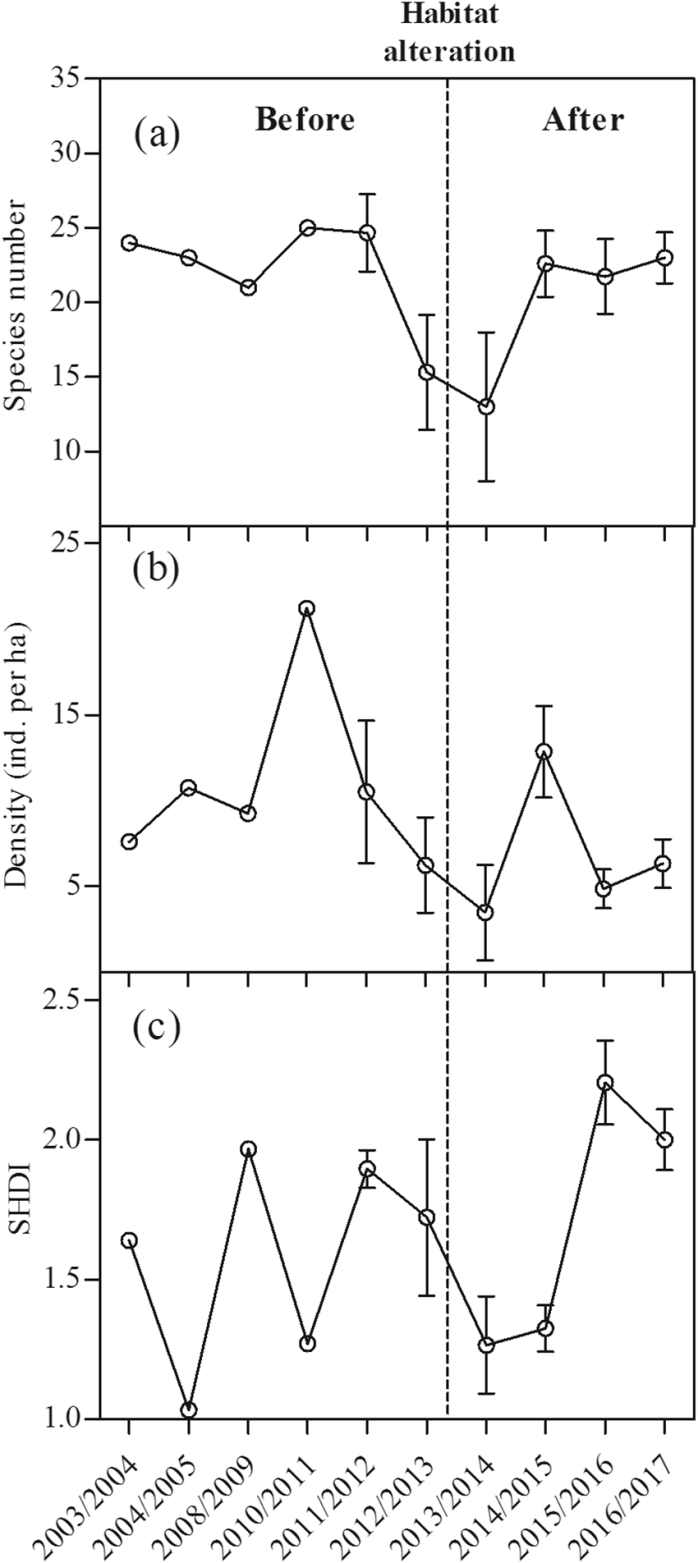
Changes in waterbird species number (**a**), density (**b**) and diversity (SHDI, **c**) in Caisang Lake from 2003/2004 to 2016/2017. Dotted lines indicate the habitat alterations.

During the study period, the abundance and richness of waterbirds in Caisang Lake exhibited both fluctuating and declining trends (Fig. 2). The highest values of species abundance and richness were both observed in the winter of 2010/2011, whereas the lowest values were both observed in the winter of 2013/2014 (Fig. 2).

**Figure 3:**
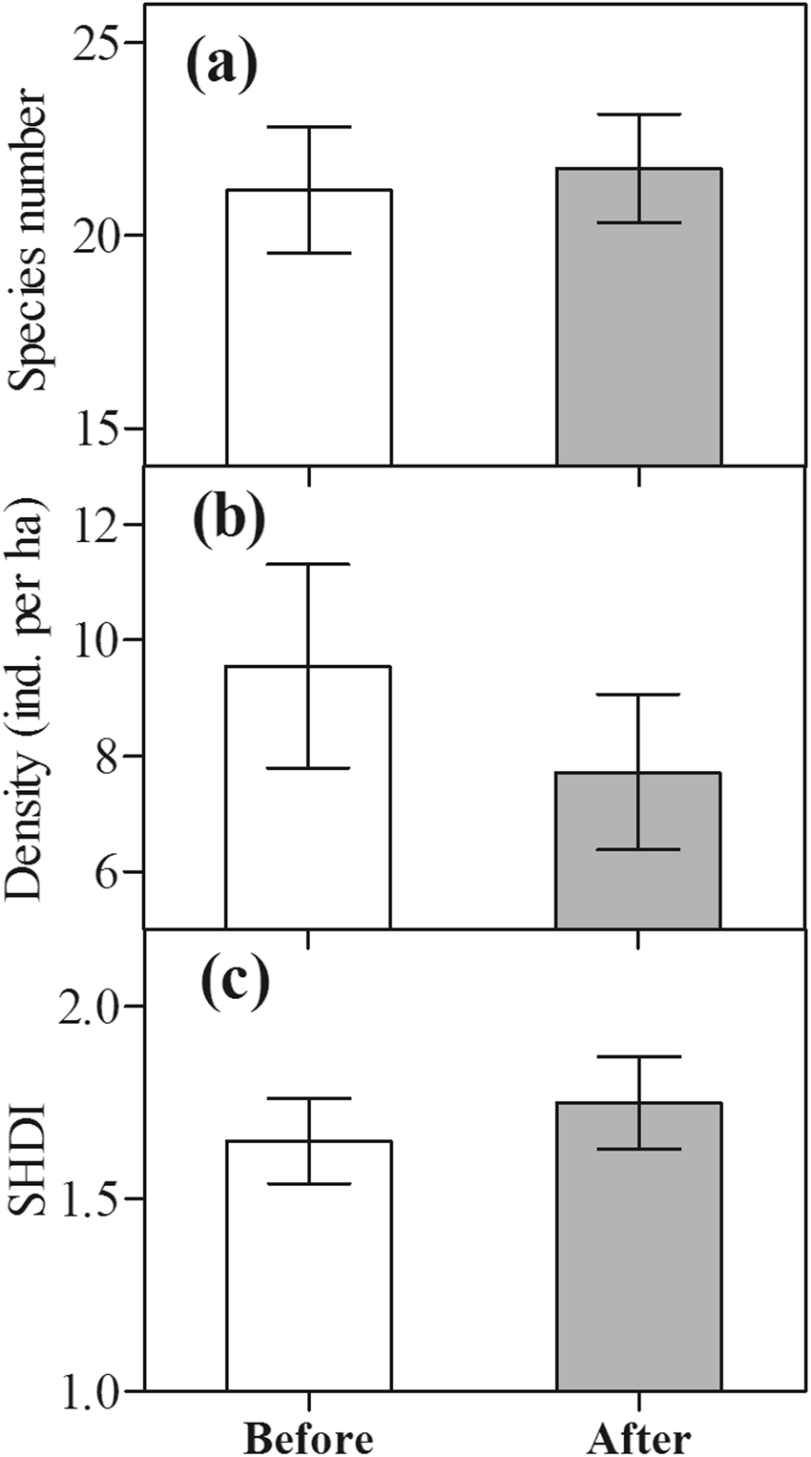
Changes in waterbird species number (**a**), density (**b**) and diversity (SHDI, **c**) before and after habitat alterations. Error bars represent standard error (SE). Statistically significant values are represented by asterisks: *p < 0.05, **p < 0.01.

At the community level, changes in total species number, density, and diversity (SHDI) of waterbirds all fluctuated from 2003/2004 to 2016/2017 in Caisang Lake (Fig. 2). The density of waterbirds exhibited a declining trend, but the species number and the diversity (SHDI) of waterbirds both appeared to increase after habitat alterations (2013/2014–2016/2017) compared to before habitat alterations (2003/2004–2012/2013; Figs 2 and 3). However, these results were not significant with α = 0.05 (density, t = 0.84, df = 25, p = 0.41 > 0.05; species number, t = −0.26, df = 25, p = 0.80 > 0.05; SHDI, t = −0.59, df = 25, p = 0.56 > 0.05; Fig. 3).

**Figure 4:**
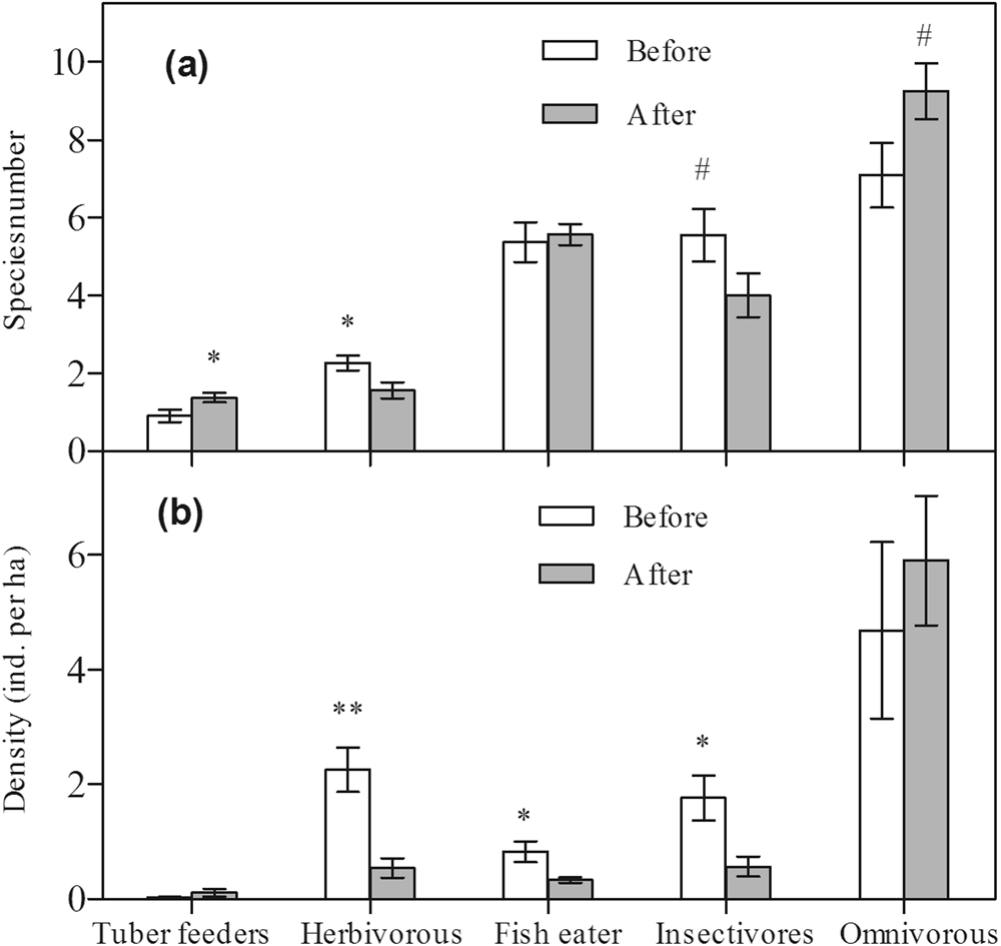
Changes in the species number (**a**) and density (**b**) of the five foraging guilds before and after habitat alterations. Error bars represent standard error (SE). Statistically significant values are represented by asterisks and the pound sign: ^#^p < 0.1, *p < 0.05, **p < 0.01.

Notably, the waterbird community composition exhibited significant changes that appeared to be foraging guild-specific; this was indicated by the changes in the species number and the densities of five foraging guilds (Fig. 4). At foraging guild level, after habitat alterations, the species number of herbivores exhibited a significantly declining trend whereas insectivores showed a nearly-significant declining trend (herbivores, t = 2.41, df = 25, p = 0.02 < 0.05; insectivores, t = 1.75, df = 25, p = 0.09 > 0.05; Fig. 4a). In contrast, after habitat alterations, tuber feeders exhibited a significantly increasing trend whereas omnivores showed a nearly-significant increasing trend (tuber feeders, t = −2.31, df = 25, p = 0.03 < 0.05; omnivorous, t = −1.95, df = 25, p = 0.06 > 0.05; Fig. 4a). Densities of three of the five waterbird guilds exhibited significant declining trends after habitat alterations (herbivores, t = 4.05, df = 25, p = 0.001 < 0.01; fish eaters, t = 2.56, df = 25, p = 0.03 < 0.05; insectivores, t = 2.34, df = 25, p = 0.04 < 0.05; Fig. 4b).

**Figure 5:**
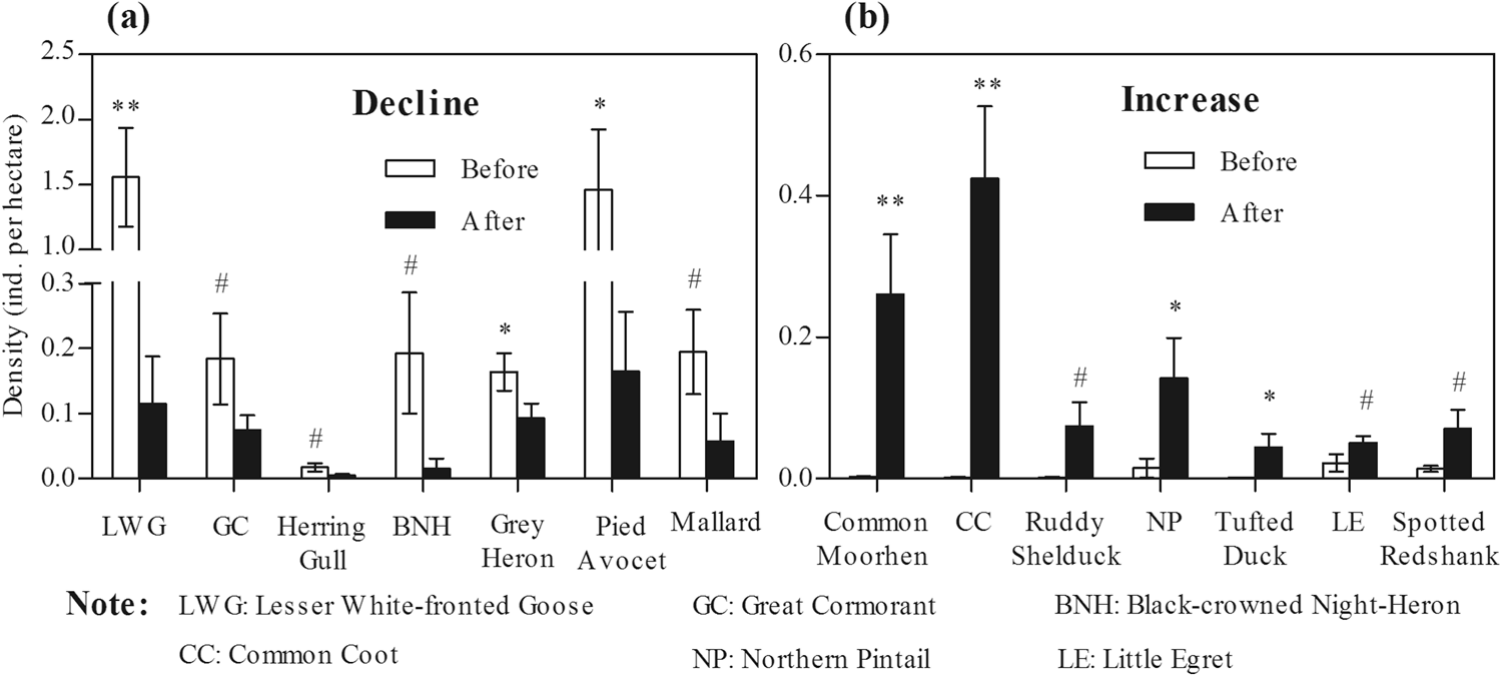
Changes in the density of individual species whose densities were significantly different before and after habitat alterations. Error bars represent standard error (SE). Statistically significant values were presented by asterisks and pound: ^#^p < 0.1, *p < 0.05, **p < 0.01.

Changes in waterbird populations after habitat alterations appear to be species-specific responses. Among the 52 species observed, 32 species (60.78% of the total species) showed declining trends (negative changes) in density, and 20 species (39.22% of the total species) showed increasing trends (positive changes, Table S1). Remarkably, densities of the lesser white-fronted goose *Anser erythropus* (t = 4.449, df = 25, p < 0.001), the grey heron *Ardea cinerea* (t = 2.142, df = 25, p = 0.042 < 0.05), and the pied avocet *Recurvirostra avosetta* (t = 2.729, df = 25, p = 0.02 < 0.05) significantly decreased after habitat alterations (Fig. 5a and Table S1). Similarly, densities of the great cormorant *Phalacrocorax carbo* (t = 1.719, df = 25, p = 0.099 < 0.1), the herring gull *Larus argentatus* (t = 1.846, df = 25, p = 0.091 < 0.1), the black-crowned night-heron *Nycticorax nycticorax* (t = 1.881, df = 25, p = 0.088 < 0.1) and the mallard *Anas platyrhynchos* (t = 1.839, df = 25, p = 0.078 < 0.1) also decreased after habitat alterations, but these were not statistically significant decreases (unless a significance level of 0.1 is considered; Fig. 5a and Table S1). In contrast, densities of the common moorhen *Gallinula chloropus* (t = −3.045, df = 25, p = 0.008 < 0.01), the common coot *Fulica atra* (t = −4.143, df = 25, p = 0.001 < 0.01), the northern pintail *Anas acuta* (t = −2.206, df = 25, p = 0.042 < 0.05), and the tufted duck *Aythya fuligula* (t = −2.229, df = 25, p = 0.041 < 0.05) significantly increased after habitat alterations (Fig. 5b and Table S1). Similarly, densities of the ruddy shelduck *Tadorna ferruginea* (t = −2.107, df = 25, p = 0.052 < 0.1), the little egret *Egretta garzetta* (t = −1.84, df = 25, p = 0.078 < 0.1) and the spotted redshank *Tringa erythropus* (t = −2.111, df = 25, p = 0.051 < 0.1) also increased, but these increases were not statistically significant (unless a 0.1 significance level is considered; Fig. 5b and Table S1). Population trends (declines and increases) were also affected by foraging guilds. Specifically, tuber feeders, herbivores, fish eaters, and insectivores were more likely to decline (respective species that disappeared per guild after habitat alteration: 2 species, 66.67%; 4 species, 80%; 10 species, 90.91%; and 12 species, 80%; Table S1), whereas omnivores were more likely to increase (13 species appeared after habitat modification, accounting for 76.47% of the total number of omnivorous species, whereas 4 omnivorous species disappeared, accounting for 23.53%; Table S1).

### Driving Factors Explaining Waterbird Population Dynamics

Stepwise regression analysis was then used to evaluate the relationships between waterbird population dynamics (at the community level, foraging guild level, and species level) and the environmental variables in the study period at Caisang Lake. The areas of mudflats, vegetation, water, and lotus, the growth status of vegetation (NDVI), and the total rainfall were generally the most important variables that explain waterbird population dynamics in Caisang Lake (Table 1). Specifically, the mudflat area was positively related to the following population dynamics: species diversity (SHDI) at the community level, the density of insectivores at the foraging guild level, and the densities of the black-crowned night-heron, the pied avocet, and the spotted redshank at the species level. In contrast, mudflat area was negatively related to the density of omnivores at the foraging guild level. The vegetation area was positively related to the density of herbivores at the species level. The growth status of vegetation (NDVI) was positively related to the densities of the lesser white-fronted goose and the mallard at the species level. The water area was positively related to the density of the grey heron at the species level. The lotus area was positively related to the density of the common moorhen at the species level. The climatic variable rainfall was negatively related to the densities of fish eaters at the foraging guild level and the herring gull at the species level.

**Table 1:** Results of the stepwise linear regression of waterbird population dynamics and environmental variables in Caisang Lake.

Species	Environment	Coefficient	Constant	Adjusted R^2^	F	p
SHDI	Mudflat area^‡^	1.386	−1.15	0.495	7.87	0.031
Herbivores^†^	Vegetation area^‡^	2.154	−3.291	0.554	9.681	0.021
Fish eaters^†^	Rainfall^‡^	−0.946	2.845	0.512	8.35	0.028
Insectivores^†^	Mudflat area^‡^	3.661	−6.377	0.765	23.844	0.003
Omnivores^†^	Mudflat area^‡^	−5.051	12.559	0.399	5.65	0.055
Lesser White-fronted Goose^†^	NDVI	21.665	−2.704	0.527	11.026	0.011
Herring Gull^†^	Rainfall^‡^	−0.081	0.191	0.387	6.055	0.043
Black-crowned Night-Heron^†^	Mudflat area^‡^	0.572	−1.108	0.249	3.651	0.098
Grey Heron^†^	Water area^‡^	0.274	−0.517	0.347	5.256	0.056
Pied Avocet^†^	Mudflat area^‡^	5.981	−11.108	0.421	6.788	0.037
Mallard^†^	NDVI	3.614	−0.424	0.313	5.097	0.054
Common Moorhen^†^	Lotus area^‡^	0.475	−0.721	0.34	5.128	0.058
Spotted Redshank^†^	Mudflat area^‡^	0.043	−0.076	0.362	4.542	0.065

^†^Represents density data. ^‡^Indicates data that has been log transformed.

## Discussion

Waterbirds are often monitored and used as biological indicators to assess the health of wetland ecosystems. Long-term changes in waterbird species and communities can be used to determine the integrity of wetland ecosystems^31,32^ and to inform wetland management policy and strategy^33–35^. In the present study, which evaluates data from 10 winter seasons between 2003/2004 and 2016/2017 at the wetlands of Caisang Lake, we demonstrate that wintering waterbird populations were sensitive to the habitat alterations that occurred in 2013/2014. In particular, the habitat alterations strongly affected the total number of species, the densities of species from five foraging guilds, and the densities of key waterbird species (Figs 4 and 5). Importantly, the present study also highlights the key environmental factors that contributed to the changes in waterbird populations. One probable cause is the drastic habitat alterations that occurred in Caisang Lake in 2013/2014; this is verified by the strong positive or negative relationships between the waterbird population variables and the environmental variables (Table 1).

At the community level, the total species number, density, and diversity (SHDI) of waterbirds exhibited no significant differences before and after habitat alterations in Caisang Lake (Fig. 3). This indicates that habitat alterations due to planting lotus and culturing crab might have different effects on particular species or foraging guilds due to their distinct feeding requirements^18^, thereby leading to no significant changes in the overall species number, density, or diversity or increase in the species number and the species diversity (SHDI) of waterbirds at the community level in Caisang Lake (Fig. 3). The waterbird community composition exhibited significant changes; responses appeared to be specific to foraging guilds and species (Figs 4 and 5). Altering the habitat by planting lotus and culturing crabs had specific impacts on species, as well as their corresponding foraging guilds^8,18,36,37^, depending on their different feeding requirements.

The area of vegetation, which was dominated by the spike rush *Eleocharis* sp. and grasses of the genus *Alopecurus*, was the major foraging habitat for herbivorous waterbird species^21^ as well as some omnivorous species in Caisang Lake. In the present study, changes in the density of herbivores exhibited positive linear correlations with changes in the vegetation area (Table 1), which sharply reduced after habitat alteration (Fig. 1). Therefore, the drastic decline in the vegetation area might have contributed to the sharp decline in the density of herbivores. Moreover, the densities of the lesser white-fronted goose and the mallard were both strongly positively related to the growth status (NDVI) of vegetation (Table 1), although not significant (Fig. 1); thus, we can infer that the decline in the growth status of the vegetation (food availability), as caused by the habitat alteration, might be the primary reason leading to the sharp decline in herbivores, especially for the lesser white-fronted goose and the mallard (Fig. 5).

The mudflat habitat was also crucial for wintering waterbirds in Caisang Lake, as indicated by the strong positive and negative correlations between the changes in the waterbird population and the mudflat area (Table 1). The mudflat habitat includes shallow water (<20 cm) that is rich with invertebrates, juvenile fish, and shrimp; these invertebrates are the main food resources for insectivores, fish eaters, and some omnivorous waterbirds. Wetlands are more productive, especially when shallow (<20 cm)^38^, where invertebrates, juvenile fish, and shrimp concentrate; the concentrations contribute to high productivity^39^, as they provide more foraging habitat for waterbirds^40^. In the present study, the positive correlation of SHDI and the mudflat area suggested that an increase in the area of mudflat habitat would maintain a high level of waterbird diversity in Caisang Lake (Table 1). Insectivores feed almost exclusively on invertebrates in mudflat and shallow water habitats^18,41^. Fish eaters, in particular Ardeidae (e.g. the black-crowned night-heron), feed almost entirely on juvenile fish and shrimp in shallow water habitats^18,36^. Omnivores feed on a variety of aquatic plant seeds and material and invertebrates^42^. Large areas of mudflats mean a richer food source for such waterbird guilds. In the present study, mudflat area was positively related to the density of insectivores at the foraging guild level and the pied avocet, the spotted redshank, and the black-crowned night-heron at the species level (Table 1). The mudflat area was drastically reduced due to the large area of lotus planted during habitat alteration (Fig. 1), which has possibly resulted in a shortage of invertebrates in such habitats; if so, this would logically lead to observed declines in the density of insectivores and fish eaters at the foraging guild level, as well as declines in the densities of the pied avocet, the spotted redshank, and the black-crowned night-heron at the species level (Fig. 5). Unexpectedly, the density of omnivores responded negatively to the changes in the mudflat area in Caisang Lake (i.e. when mudflat areas decreased, omnivores increased; Table 1). This seems to imply that omnivores could maintain a high density (Fig. 3) despite the sharp decline in mudflat area after 2013/2014 (Fig. 1). However, most of the omnivorous species, especially approximately 10 thousand ducks and thousands of common coot, were observed foraging in the lotus ponds rather than the mudflat and shallow water habitats.

The water habitat includes shallow water and has a rich abundance of fish that is the main food resource for fish eaters, e.g. the grey heron^43^. Previous studies indicated that the water area was crucial for fish eaters, e.g. Ardeidae, Laridae, and cormorants, which all feed almost exclusively on fish^18,20,36,42^. However, the present study revealed that only the grey heron was positively affected by the size of the water area (Table 1). Caisang Lake was previously used for cultivating fish, before the habitat alterations in 2013/2014; however, large areas were used for cultivating crab after the habitat alterations. Such dramatic changes would lead to a significant decline in density of fish; thus, this might be the most probable cause of the sharp decline in the density of fish eaters, especially the grey heron, the black-crowned night-heron, the great cormorant, and the herring gull (Fig. 5; Table S1).

Lotus (wild lotus) already existed in Caisang Lake before the habitat was altered in 2013/2014; however, the initial area covered by lotus was small (<50 ha). Some waterbirds, particularly the common moorhen and the common coot, were observed foraging in wild lotus habitat before the habitat alterations. A large area of lotus (approximately 300 ha) was planted in Caisang Lake in 2013/2014, thereby replacing the native vegetation. After the lotus was planted, four guilds of waterbirds, i.e. tuber feeders, herbivores, fish eaters, and insectivores, disappeared from lotus ponds. This is possibly due to the shortage of foods. However, omnivorous waterbirds, including approximately 10 thousand ducks and thousands of the common coot, were observed foraging in the lotus ponds (Table S1). Moreover, the common coot was significantly positively related with the size of the lotus area (Table 1). The habitat alterations caused by planting lotus were negative for tuber feeders, herbivores, insectivores and fish eaters, but positive for omnivores as the alterations directly impacted food resources (tuber and root of lotus, and aquatic plant and material, e.g. phytoplankton).

Climatic variables (e.g. temperature and rainfall) also played important roles in waterbird population dynamics^8,37^, which should not be neglected. In addition to temperature, rainfall was a crucial factor affecting waterbird populations in Caisang Lake, as inferred by the negative correlation between the density of fish eaters and rainfall (Table 1). This might be due to the large area of shallow water habitat when rainfall was low, where juvenile fish and shrimp concentrate; this contributes to high food availability^39^ and provides a more suitable foraging habitat for fish eaters^40^.

Other variables, though not analysed in this study, might also influence the abundances and distributions of wintering waterbirds. Importantly, previous studies have suggested that human disturbances account for important changes in waterbird populations^9^. In 2013/2014, approximately 6 km of road was constructed throughout the wetlands of Caisang Lake; this would undoubtedly threaten the wintering waterbirds. Therefore, human disturbance is an important consideration when evaluating wintering waterbird community patterns and is an area that requires future investigation.

Our results indicate that wintering waterbird populations exhibited significant changes after habitat alterations in Caisang Lake; these changes appeared to be foraging guild-specific and species-specific. Waterbird population changes were significantly affected by habitat changes due to the planting of lotus ponds and crab cultivation. Drastic changes in the areas of vegetation, mudflat, water, and lotus habitats, as well as the growth status of the vegetation (NDVI) and rainfall, were environmental driving factors that affected waterbird population dynamics. We suggest that several conservation measures be quickly implemented as part of the management plan for human-modified wetlands. First, the area covered by lotus should not be increased, but rather it would ideally be reduced. Secondly, and more importantly, in order to reduce the impact of harmful human disturbance on the wintering waterbirds, lotus should not be harvested during the winter season. Effective measures, e.g. transforming a certain area of the lotus ponds back to the original types of wetlands (i.e. vegetation, mudflat, and water), should be undertaken to increase the food availability for wintering waterbirds, especially for those whose densities sharply declined (e.g. herbivore, insectivores, and fish eaters). At least part of the water area should not be used for culturing crab but rather for culturing fish, thus providing sufficient food resources for waterbirds that feed on fish and shrimp (e.g. fish eaters). In addition, roads throughout the Caisang Lake should be removed to minimize human disturbance to the wintering waterbirds.

## Methods

### Study area

**Figure 6:**
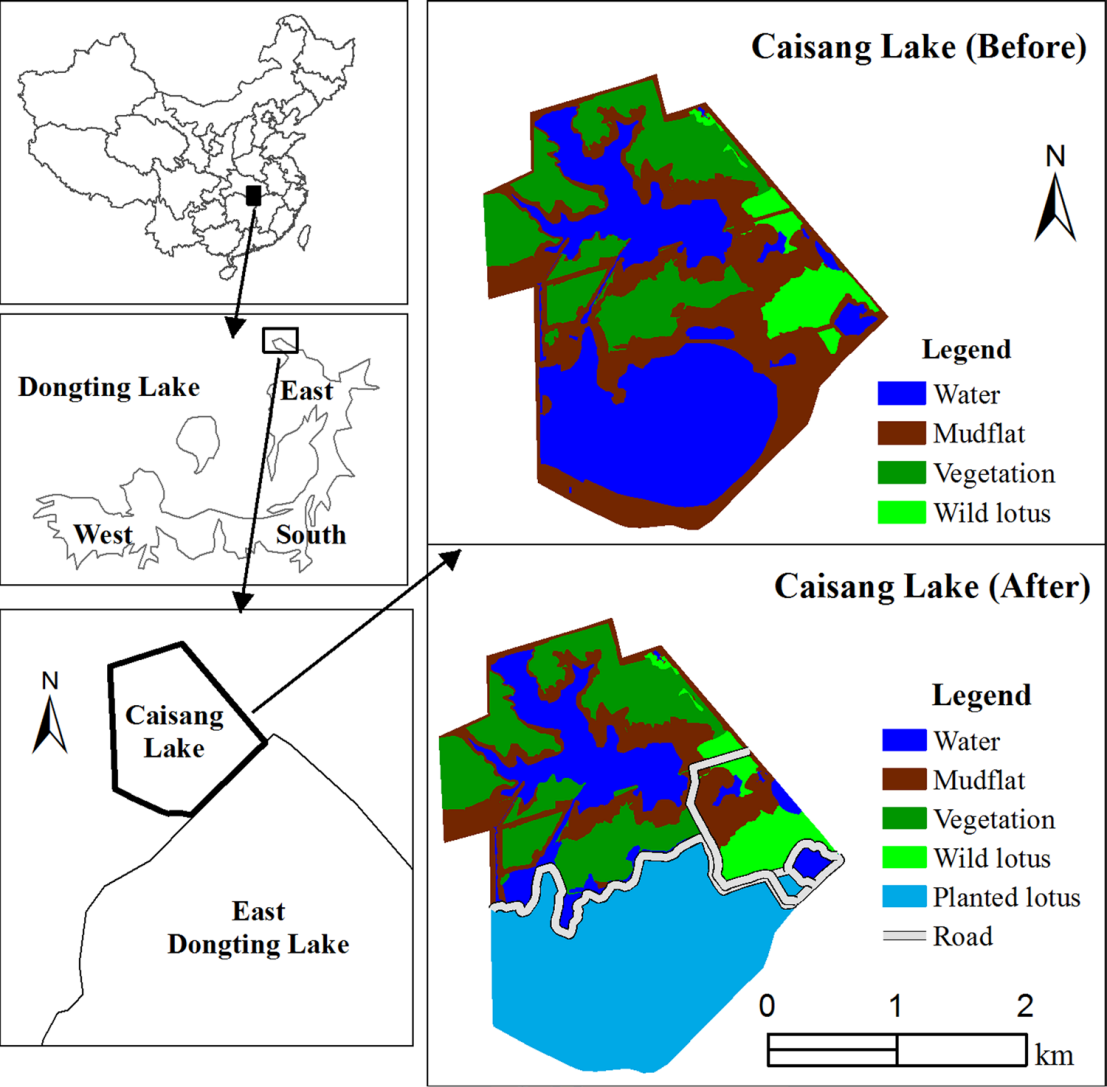
Location of the study area at Caisang Lake. The map was created using ArcGIS 10.0 (http://www.esri.com/software/arcgis/arcgis-for-desktop).

Dongting Lake is the second largest freshwater lake in China, consisting of three parts: East, South, and West (Fig. 6). The substantial biodiversity of Dongting Lake has led to its recognition as one of the 200 global conservation priority eco-regions in the world^4^. A northwestern part, Caisang Lake, was selected as the study area (Fig. 6) because it is an important alternative habitat for migratory wintering waterbirds, especially when the habitat quality has decreased in the natural wetlands of East Dongting Lake^7,21,22^. Caisang Lake comprises 700 ha of human-modified wetlands. Before 2014, the major habitat types in Caisang Lake included recessional grasslands (dominated by the spike rush *Eleocharis* sp. and grasses of the genus *Alopecurus*), mudflats, and fish ponds. In January 2013 and 2014, an area of nearly 300 ha of lotus (*Nelumbo nucifera*) ponds was constructed by building pond levees and removing all prior vegetation in the ponds; meanwhile, the remaining lake (minus the lotus ponds) was used to culture Chinese mitten crab (*Eriocheir sinensis*) instead of fish, which had been cultivated prior (Fig. 6). Therefore, after drastic habitat alterations in 2013/2014, the major habitat types became recessional grasslands, mudflats (both areas were severely diminished), water (e.g. crab pond), and lotus pond. Moreover, roads (i.e. pond levees, approximately 6 km) were constructed throughout the Caisang Lake area in 2013/2014 (Fig. 6).

### Waterbird surveys

Waterbird survey data from 2003/2004–2013/2014 were acquired from the management office of the East Dongting Lake Nature Reserve. All surveys covered all types of habitats in the study areas (Fig. 6) and were carried out in one day. Each survey started 1 h after sunrise and lasted 4–5 h. Two to three investigators counted all individual waterbirds using 10 × 42 binoculars and 20 × to 60× spotting scopes by walking along the fixed line transect. We resurveyed waterbirds in the winters of 2014/2015–2016/2017 in the same areas as previous waterbird surveys in the winters of 2003/2004–2013/2014, including areas that had been converted to planted lotus ponds in artificial wetlands. We used the same protocols that had been used for all previous surveys between 2002/2003 and 2013/2014. A total of 27 days of waterbird surveys were carried out during the study period. Specifically, surveys were conducted once per wintering season (conducted in January) of 2003/2004, 2004/2005, 2008/2009, and 2009/2010, three times per year (conducted in November, January, and March) between 2010/2011 and 2015/2016, and five times in the wintering season (once per month from November to March) of 2016/2017.

Species number, density, and diversity of all observed species were used to estimate the waterbird population dynamics at the community level. Similar to the study by Armitage *et al*.^44^, the Shannon-Weiner diversity index (SHDI) was calculated to describe the diversity of waterbirds in each wintering season between 2003/2004 and 2016/2017, according to $SHDI=-\sum ({p}_{i})(\mathrm{ln}\,{p}_{i})$, where *p*_i_ is the proportion of waterbirds that belong to the *i*th species^45^. Due to the distinct feeding requirements of specific waterbird assemblages, wintering waterbird species were aggregated into five functional guilds according to their feeding habits^8,36,37^: tuber feeders, herbivores, fish eaters, insectivores, and omnivores (Table 2). All the observed wintering waterbird species and their associated guilds are listed in Appendix S1. Species numbers and densities of the five foraging guilds were used to evaluate the waterbird population dynamics at the foraging guild level. Densities of individual species were used to evaluate the waterbird population dynamics at the species level.

**Table 2:** Classification of foraging guilds of wintering waterbirds in East Dongting Lake.

Foraging guild	Number of species	Foraging source^8,36,37^
Tuber feeders	3	Aquatic or terrestrial tubers
Herbivores	5	Tender leaves of Sedge and Gramineae
Fish eaters	11	Fish and shrimp in shallow and deep water
Insectivores	15	Aquatic insects and molluscs in mudflats, crustaceans
Omnivores	18	Tender leaves, seeds, insects, fish, shrimp, etc.

### Environmental variables

Seven environmental variables were chosen to assess the influence of environment changes on waterbird population dynamics before and after habitat alteration in Caisang Lake between 2003/2004 and 2015/2016. The seven variables included five habitat variables: water area, mudflat area, vegetation area, lotus area (including wild and planted lotus), growth status of vegetation (NDVI). We also included two climatic variables: rainfall and cumulative temperature (BioT). The five habitat variables were extracted from Landsat TM/ETM+/OLI images (30 m resolution) from between 2003/2004 and 2016/2017, which were available and downloaded from the Earth Resources Observation and Science Center (http://glovis.usgs.gov/). The periods of data acquisition were matched according to the dates of the waterbird surveys in each wintering season. Twenty-four satellite images were used, including twelve Landsat TM images, five Landsat ETM+images, and seven Landsat 8 OLI images. The satellite images were chosen and processed based on the framework by Xie *et al*.^6^. A supervised method was used for the classification of five habitat variables. Classification accuracy was evaluated using a standard error matrix (confusion matrix)^29^ that reported overall classification accuracies. Kappa chance correction statistics were prepared for each image to determine the accuracy of the classifications. Similar to the study by Wang *et al*.^8^, rainfall was calculated using the sum of daily rainfall, whereas BioT was calculated using mean daily temperatures greater than 5 °C in each winter season. Data on daily rainfall and temperature were recorded at Yueyang weather station during the study period and were acquired from Weather Underground (https://www.wunderground.com).

### Statistical analysis

D’Agostino-Pearson omnibus tests^46^ were used to analyse whether the waterbird variables (the total number of species, the total density, the SHDI of all waterbirds, the densities of five foraging guilds, and the densities of each waterbird species) and the environmental variables (water area, mudflat area, vegetation area, lotus area, NDVI, rainfall, and BioT) were normally distributed. Results of these tests indicated that all variables passed normality tests (all p > 0.05).

We analysed temporal changes in seven environmental variables before (2003/2004 to 2012/2013) and after habitat alteration (2013/2014 to 2016/2017) in Caisang lake via independent *t* test.

Because waterbird communities might differ in their utilization of Caisang lake before and after habitat alteration, we compared the total number of species, the total density, and the species diversity (SHDI) at the community level, the densities of five guilds at foraging guild level, and the densities of individual species at the species level in Caisang Lake before and after habitat alterations via independent *t* test.

To estimate the relative importance of environmental variables in explaining waterbird population dynamics in Caisang Lake, stepwise linear regression analyses were used to examine the correlations between environmental variables and waterbird population dynamics at the community, foraging guild, and species levels.

Normality tests were performed using GraphPad Prism version 6.0 for Windows. Independent t test and all stepwise linear regression analyses were performed using the Software Package for Social Statistics (IBM SPSS Statistics Version 21.0).

## Electronic supplementary material

Supplementary Dataset 1
